# Electron microscopy combined with spatial analysis: quantitative mapping of the nano-assemblies of plasma membrane-associating proteins and lipids

**DOI:** 10.1007/s41048-018-0060-4

**Published:** 2018-07-25

**Authors:** Yong Zhou, John F. Hancock

**Affiliations:** Department of Integrative Biology and Pharmacology, The University of Texas Health Science Center at Houston, Houston, TX 77030 USA

**Keywords:** Electron microscopy, Spatial distribution, Lipid-anchored proteins, Nanoclustering, Signal transduction, Ripley’s *K*-function

## Abstract

The plasma membrane (PM) is a complex environment consisting of > 700 species of lipids and many different types of membrane-associating proteins. These lipids and membrane proteins are distributed heterogeneously into nanometer-sized domains, called nanoclusters. The lateral spatial segregation in the PM gives rise to different curvature and lipid composition, which determines the efficiency of effector binding and signal transmission. Here, we describe an electron microscopy (EM)-spatial mapping technique to quantify the extent of nanoclusters formation in the PM. The nano-assemblies in the PM are quantified via expressing the GFP-tagged proteins or lipid-binding domains in the cells, which are then immunolabeled with the gold nanoparticles pre-coupled to the anti-GFP antibody. The gold nanoparticles are visualized via the transmission EM at high magnification. The statistical analysis of the Ripley’s *K*-function calculates the spatial distribution of the gold nanoparticles. Important spatial parameters, such as the extent of nanoclustering, the clustered fraction, the number of proteins per cluster, the optimal size of a nanocluster, and the number of proteins localized to the PM, can be calculated. Further detailed aggregation pattern, such as the populations of monomers, dimers, trimers, and higher ordered oligomers, can also be extracted from the spatial analysis. The EM-bivariate analysis quantifies the extent of co-localization between two different components in the PM and provides key information on the protein–protein and the protein–lipid interactions over a long-distance scale from 8 to 240 nm.

## Introduction

The plasma membrane (PM) is a highly complex nano- and micro-environment with ~700 different species of lipids and many membrane-associating proteins (Simons and Toomre 2000). Biophysical studies using synthetic model bilayers, isolated native giant plasma membrane vesicles (GPMVs) and intact PM sheets, fixed cells and intact tissues all demonstrate that the lipids and the proteins laterally segregate into nanoclusters with diameters from < 20 nm to a few hundred nanometers (Baumgart *et al*. 2003; Levental *et al*. 2011; Plowman *et al*. 2005; Prior *et al*. 2003a; Veatch and Keller 2002). This kind of nanoclustering gives rise to a vastly complex environment in the PM with each membrane protein possessing distinct lipid environment close to its vicinity (Zhou *et al*. 2014, 2015, 2017). The purpose of such complex lateral spatial segregation is still not clear. However, as many downstream effectors contain the specific lipid-binding domains, such as the C2 and the PH domains, it is apparent that the PM nanoclustering, along with the distinct lipid composition, is critical to effector recruitment and signal transmission (Zhou *et al*. 2015, 2017).

It is difficult to characterize the lateral spatial distribution of the lipids and the membrane proteins. We utilized a quantitative imaging technique of electron microscopy (EM) combined with spatial mapping to mathematically determine the extent of nanoclustering of the lipids or the proteins in the PM (Prior *et al*. 2003a, b). To determine the univariate nanoclustering of a membrane protein, we ectopically expressed the GFP-tagged protein of interest in the cells and attach the intact PM sheets to the copper EM grids coated with pioloform and poly-l-lysine. The location of the GFP-tagged protein was then marked by incubating the PM sheets with a solution containing 4.5-nm gold nanoparticles pre-coupled to anti-GFP antibody. The gold particle distribution was then imaged and digitized using the transmission EM. The coordinates of the gold particles were assigned using ImageJ, and the extent of nanoclustering of the gold particles was calculated using the statistical analysis of Ripley’s *K*-function. To quantify the univariate nanoclustering of a PM lipid, we expressed a GFP-tagged specific lipid-binding motif (Zhou *et al*. 2014, 2015, 2017). For example, we expressed GFP-LactC2 to label the location of phosphatidylserine (PS) in the PM since the C2 domain specifically binds to PS (Zhou *et al*. 2014, 2015, 2017). The location and the spatial distribution of PS can then be visualized with the gold particle labeling and the Ripley’s *K*-function analysis. The same technique can also be applied to study the co-localization between two distinct components in the PM (Zhou *et al*. 2014, 2015, 2017). We expressed one component tagged with GFP and another tagged with RFP and label the components with 6-nm gold particles coupled to anti-GFP and 2-nm gold particles linked to anti-RFP (Prior *et al*. 2003a; Zhou *et al*. 2012, 2014, 2015, 2017). The co-localization between the larger 6-nm gold and the smaller 2-nm gold can be calculated using the bivariate *K*-function. Using the bivariate technique, we can map the detailed protein–protein and protein–lipid spatial nano-assembly in the PM. Combining the univariate and the bivariate analyses, we can deduce the complex spatial environment in the PM: how the extent of nanoclustering of a certain membrane protein along with its complex lipid composition influence the downstream effector binding and the signaling activities (Zhou *et al*. 2014, 2015, 2017).

## Protocol

### Preparation of reagents for EM gold-labeling

#### Solutions

1. 1% trisodium citrate: 0.5 g of sodium citrate dissolved in 50 ml of deionized water, stored at room temperature.
2. 1% tannic acid: 0.5 g Aleppo tannin dissolved in 50 ml of deionized water, stored at room temperature.
3. 25 mmol/L potassium carbonate: 0.173 g of potassium carbonate dissolved in deionized water, stored at room temperature.
4. Reducing solution: 1% trisodium citrate, 1% tannic acid, 25 mmol/L potassium carbonate and deionized water for a final volume of 10 ml. The amount of trisodium citrate, tannic acid, and potassium carbonate varies for different-sized gold particles (see Table 1).

**Table 1:** The amount of tannic acid and potassium carbonate for different-sized gold particles

	Gold nanoparticle size (nm)					
	2–3	4	4.5	5.5	6	7.5
1% tannic acid (ml)	2.5	1.25	0.75	0.5	0.25	0.13
25 mmol/l K_2_CO_3_ (ml)	2.5	1.25	0.75	0.5	0.25	0.13
5. 1% gold chloride (stock): 1 g of gold chloride dissolved in 100 ml deionized water, stored at 4 °C for >2 years.
6. Gold solution (for making the gold nanoparticles): 0.5 ml of 1% gold chloride dissolved in deionized water.
7. 10% BSA: 2 g of BSA dissolved in 20 ml of PBS, stored at 4 °C.
8. 0.1% BSA in PBS: 0.5 ml of 10% BSA diluted in 50 ml PBS.
9. Glycerol gradient (10%–40%): varying amount of glycerol and 50 μl BSA dissolved in PBS for a final volume of 5 ml (see Table 2).

**Table 2:** Varying amount of glycerol and 10% BSA dissolved in PBS for a final volume of 5 ml

	% Glycerol						
	10	15	20	25	30	35	40
Glycerol (ml)	0.5	0.75	1	1.25	1.5	1.75	2
10% BSA (ml)	0.05	0.05	0.05	0.05	0.05	0.05	0.05
PBS (ml)	4.45	4.2	3.95	3.7	3.45	3.2	2.95
10. 2% methyl cellulose: 2 g of methyl cellulose dissolved in 98 ml of deionized water, stored at 4 °C.
11. 10× KOAc buffer (stock solution): 11.92 g HEPES, 22.58 g potassium acetate, and 1.02 g magnesium chloride dissolved in 180 ml deionized water, stock stored at −20 °C while the working solution stored at 4 °C.
12. Fixative: 2 ml of 16% paraformaldehyde, 32 μl of 25% glutaraldehyde, and 0.8 ml of 10× KOAc stock combined with 5.17 ml of deionized water, stored at 4 °C.
13. 25 mmol/L glycine: 93.8 mg glycine dissolved in 50 ml of PBS. Aliquots of 1 ml stored at 4 °C.
14. 10% fish skin gelatin: 3 ml of 45% fish skin gelatin solution combined with 11.5 ml of PBS and stored at 4 °C.
15. Blocking solution: 100 μl of 10% fish skin gelatin and 100 μl of 10% BSA combined with 9.6 ml of 1× KOAc buffer (diluted from the 10× stock).
16. 3% uranyl acetate: 1.5 g of uranyl acetate dissolved in 50 ml of deionized water and stored at 4 °C.

### Preparation of antibody coupled gold nanoparticles

1. Add 10 ml of reducing solution (see section “Solutions” (4)) and 40 ml of gold solution (see section "Solutions" (6)) in separate flasks and heat to ~60 °C. The amount of solutions added differs based on the size of the gold particles (see Table 1). We typically make 4.5-nm gold particles.
2. Combine two solutions once temperature reaches 60 °C.
3. Boil the combined solution for 5 min with constant stirring. Proper colloid formation should yield a maroon color solution.
4. Cool down the solution by leaving the flask on ice.
5. Adjust the pH to ~8.5 using pH paper only (gold solution can damage pH electrodes). We typically use 0.5 mol/L NaOH solution to adjust the pH. First add ~150 μl and then 10 μl increments.
6. To determine the amount of anti-GFP antibody needed, we add 0–5 μg of anti-GFP antibody (diluted to 20 μl final volume using deionized water) into 250 μl of gold solution to titrate the optimal antibody level.
7. Incubate the mix at room temperature for 5 min.
  (A) Add 100 μl of 10% NaCl and mix well.
  (B) Solutions become bluer in color as more antibodies are present. The optimal amount of the antibody (the lowest amount of antibody) is indicated by the tube that no longer changes its color to blue.
8. Calculate the amount antibody needed for the total amount of gold solution available and combine antibody with the gold solution.
9. Incubate the mix for 10 min at room temperature and add 10% BSA to the gold/antibody solution to achieve a final BSA concentration of 0.1%.
10. Centrifuge the mixed gold-antibody solution at 100,000 *g* (for 4–6 nm gold) or 120,000 *g* (for 2–3 nm gold) for 1 h at 4 °C.
11. Collect the gold-antibody complex by aspirating the loose pallet using a micropipettor. Note that the solid round pellet on the side of tube is the unbound gold and should be discarded.
12. For univariate analysis, the gold-antibody complex made here should be appropriate to use. For bivariate analysis, the requirement for accurate gold particle size is stricter. The gold particles made using above procedure should be further processed.
13. To further process, load the gold-antibody complex obtained from above procedure into 5 ml ultracentrifuge tubes containing various amount of glycerol (see Table 2).
14. Centrifuge 100,000 *g* for 6 nm gold or 120,000 *g* for 2–3 nm gold for 1 h at 4 °C.
15. Collect gold solution in the top half of the gradient.
16. To calibrate the gold size distribution, pipette 100 μl of the gold solution onto a parafilm sheet and place a pioloform- and poly-l-lysine-coated EM copper grid on the bubble. Incubate 5 min and let dry for at least 30 min before EM imaging.

### Pioloform- and poly-l-lysine coating EM grids

#### Pioloform-coating

1. Dissolve 0.4 g of pioloform in 50 ml of chloroform and shake well to mix.
2. Fill a 25-ml cylindrical separatory funnel half-full with the pioloform/chloroform solution and dip an ethanol-cleaned glass slide into the funnel.
3. Drain the pioloform/chloroform solution, leaving a thin film of pioloform on the slide.
4. Use a razor blade to cut lines along the edges of the pioloform film on the glass slide.
5. Slowly dip the glass slide into deionized water in a small bowl. If the pioloform film has been properly formed and cut, the film will slide off the glass slide and float on the water surface.
6. Carefully position copper or gold EM grids onto the pioloform film with the dull side down. Approximately 40–50 grids can be placed on a pioloform film.
7. Place a clean glass slide coated with a self-adhesive paper vertically above the pioloform film and slowly pushing down, allowing the film to coat the glass slide. Pioloform-coated grids are then left to dry overnight.

#### Poly-l-lysine coating

1. Pipette a 2-ml poly-l-lysine bubble on a sheet of parafilm. Slowly cut the pioloform-coated EM grids using fine-tipped tweezers and place on the poly-l-lysine bubble with the dull side (pioloform-coated) down.
2. Allow incubation for 5 min before washing the grids twice by placing the grids on a 2-ml deionized water bubble.
3. The poly-l-lysine-coated grids are then left to dry overnight.

### PM rip-off

PM rip-off can be performed to attach either apical PM or basolateral PM onto the grids. Copper grids are used for apical PM rip-off, while gold grids are used for basolateral PM attachment since copper is toxic to cells. The initial steps differ slightly for the two techniques.

#### Apical PM rip-off

1. Cells grown on glass coverslips in a 3.5-cm dish are washed twice with PBS. The glass coverslip with the appropriate cell confluency (80%–90%) is then placed on a piece of clean filter paper with the cell side facing up. Two pioloform- and poly-l-lysine-coated copper EM grids are then positioned on cells with the dull side down (with the pioloform- and poly-l-lysine-coated side facing the cells on the glass coverslips).
2. A rubber bung is used to press down on the EM grids to attach the apical PM to the grids.
3. Approximately 100 μl 1× KOAc solution is pipetted onto the glass coverslip around the copper EM grids, making sure not to cover the grids with KOAc solution. The surface tension of the aqueous solution will pop the grids off the glass coverslip, with only the intact apical PM sheets firmly attached to the grids and leaving the rest of the cells on the glass coverslips.

#### Basolateral PM rip-off

1. When the basolateral PM needs to be collected, cells are seeded directly over the pioloform- and poly-l-lysine-coated gold EM grids.
2. At the time of experiment, the gold EM grids are washed twice with PBS and placed on a piece of clean filter paper. Another piece of clean filter paper pre-wetted with PBS solution is then placed on the top for 10 min.
3. The wet filter paper on the top is slowly peeled off, leaving only the basal PM attached to the gold EM grids.
4. To further eliminate endomembranes, a PBS-wetted glass coverslip is placed on top of the gold EM grids.
5. A rubber bung is used to press down on the coverslip. A 100 μl 1× KOAc bubble is pipetted onto the glass coverslip around the gold EM grids, making sure not to cover the grids with KOAc solution. The surface tension of the aqueous solution will pop the grids off the glass coverslip, with only the basal PM sheets firmly attached to the grids and leaving the rest of the cells on the glass coverslips.

#### Fixing and labeling procedure

1. EM grids with flattened PM are then placed on a 50-μl fixative bubble for 10 min. Note that, the protocol from this point on is the same for both apical and basolateral PM rip-off.
2. After blotting the EM grids by position the grids on a piece of filter paper at a 45° angle, the grids are then moved to a 100 μl PBS bubble for 5 min.
3. EM grids are quenched by three consecutive 100 μl quenching bubbles (1× glycine solution diluted in 1× KOAc).
4. EM grids are then placed on a 100 μl blocking solution bubble for 20 min.
5. After blotting, the grids are then placed on a 10-μl bubble containing gold nanoparticles coupled to anti-GFP antibody for 30 min. For bivariate experiments, the grids should be incubated with large 6-nm gold coupled to anti-GFP first, blocked for another 5 min, then incubated with 2-nm small gold coupled to anti-RFP antibody.
6. After blotting, the grids are placed on five consecutive 100 μl blocking solution bubbles, each for 5 min.
7. After blotting, the grids are then placed on five consecutive 100 μl deionized water bubbles. Make sure to wash the tweezers with deionized water thoroughly between each step.
8. Move grids onto a 100 μl uranyl acetate bubble (10:1 dilution using methyl cellulose) on ice for 10 min.
9. Scoop the grids from uranyl acetate bubbles using a grid loop and blot off the excess uranyl acetate on a piece of filter paper. Allow grids to dry overnight.

### Imaging and analysis

#### Obtain and digitize images

**Fig. 1:**
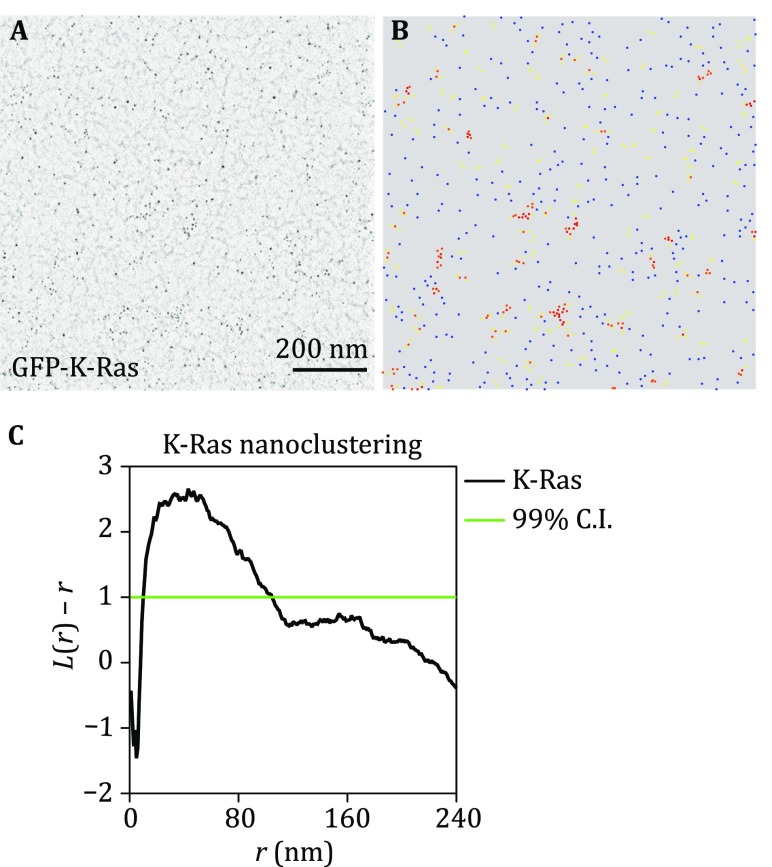
Univariate nanoclustering analysis quantifies the lateral spatial distribution of a single population of gold nanoparticles on the intact plasma membrane sheets. **A** An intact PM sheet of kidney cells ectopically expressing GFP-K-Ras was attached to an EM grid, fixed and immunolabeled with 4.5-nm gold nanoparticles. Gold distribution on the intact PM sheet was imaged using transmission EM at 100,000× magnification. **B** *x* and *y*-coordinates of the gold particles were assigned using ImageJ and were used to calculate the spatial distribution. *Blue dots* indicate monomers; *yellow dots* indicate dimers; *orange dots* indicate trimers; *red dots* indicate higher ordered oligomers. **C** The extent of nanoclustering, *L*(*r*) − *r*, was plotted against the length scale, *r*, in nanometer. *L*(*r*) − *r* values above the 99% confidence interval (99% CI) indicate statistically meaningful clustering

Obtain and digitize images of PM sheets with proper gold labeling using a transmission EM at the magnification of 100,000× (Fig. 1A).

#### Data analysis

**Fig. 2:**
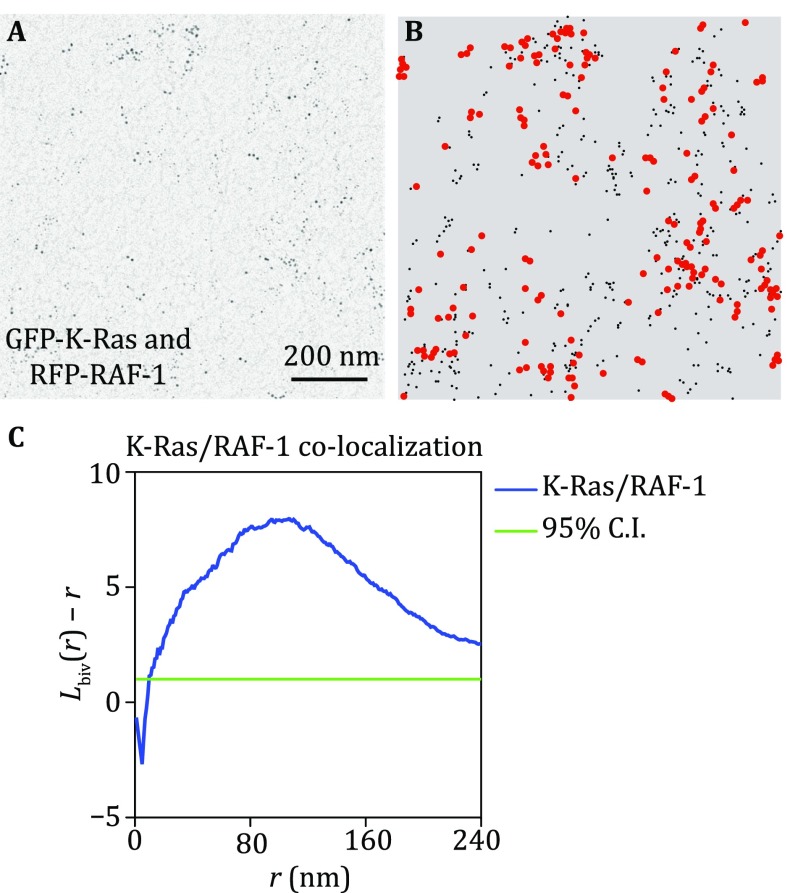
Bivariate co-clustering analysis characterizes the co-localization between two populations of gold nanoparticles. **A** An intact PM sheet of kidney cells ectopically co-expressing GFP-K-Ras and RFP-RAF-1 was attached to an EM grid, fixed and immunolabeled with 6-nm gold labeling GFP and 2-nm gold labeling RFP. Gold distribution on the intact PM sheet was imaged using transmission EM at 100,000× magnification. **B** *x* and *y*-coordinates of the gold particles were assigned using ImageJ and were used to calculate the co-localization between the two populations of gold. Smaller *black dots* indicate 2-nm gold labeling RFP; larger *red dots* indicate 6-nm gold labeling GFP. **C** The extent of co-localization, *L*_biv_(*r*) − *r*, was plotted against the length scale, *r*, in nanometer. *L*_biv_(*r*) − *r* values above the 95% confidence interval (95% CI) indicate statistically meaningful co-localization

1. Univariate *K*-function: the spatial distribution of a single species labeled with 4.5-nm gold coupled to anti-GFP.
  (A) Open an EM image file in ImageJ and assign the *x* and *y* coordinates to each gold particle within a selected 1 μm^2^ area (Fig. 1B). Ensure that the gold particles in the selected area distribute in the most uniform manner, where no visible pattern can be discerned by naked eye.
    i. The raw EM images saved as TIFF file is opened in the ImageJ.
    ii. A 1 μm^2^ PM sheet area is selected and cropped, which is further smoothened.
    iii. The background of the image is subtracted using a rolling ball radius of six pixels.
    iv. The gold nanoparticles are then highlighted using the Threshold function.
    v. The *x* and *y* coordinates to each gold particle are assigned using the “Analyze particle” function, with particle size range of 2–80 pixel^2^.
  (B) The clustering of gold particles is analyzed using variations of the statistical analysis of Ripley’s *K*-function (Ripley 1977) (Eqs. 1–2):
    1 $$K\left( r \right) = An^{ - 2} \mathop \sum \limits_{i \ne j} w_{ij} 1(\Vert x_{i} - x_{j} \Vert \le r)$$
    2 $$L\left( r \right) - r = \sqrt {\frac{K\left( r \right)}{\pi }} - r$$
    where *K*(*r*) describes the univariate spatial *K*-function for the gold nanoparticles within a selected PM area defined as *A*; *n* is the total number of gold particles within the PM area; *r* is the length scale originating from each gold particle; || · || indicates the Euclidean distance, with 1( · ) given a defined value of 1 if ||*x*_*i*_ − *x*_*j*_|| ≤ *r* and a value of 0 if ||*x*_*i*_ − *x*_*j*_|| > *r*; and *w*_*ij*_^−1^ is the fraction of the circumference of a circle with center at *x*_*i*_ and a radius ||*x*_*i*_ − *x*_*j*_||. *K*-function is then standardized to yield *L*(*r*) − *r*, via a 99% confidence interval (CI) estimated by Monte Carlo simulations (Fig. 1C). Typically, a minimum of 15–20 PM sheets are imaged and analyzed for each condition.
    (C) The statistical significance of differences between replicated point patterns is evaluated in bootstrap tests constructed as described (Diggle *et al*. 2000; Plowman *et al*. 2005).
    (D) The number of gold nanoparticles is also counted within the same 1 μm^2^ PM area used for the clustering analysis above. The gold number is an estimate of the level of the targeted membrane proteins on the PM.
      i. To ensure that the changing immunogold-labeling reflects a changing protein level, but not changing total expression level of the protein, Western blotting using anti-GFP antibody is usually run to validate the total expression level.
      ii. The statistical significance between the comparing sets is evaluated using the one-way ANOVA.
2. Bivariate *K*-function: co-localization of two populations of proteins/lipid-biding domains labeled by different-sized gold particles (Fig. 2A, 6-nm gold coupled to anti-GFP and 2-nm gold linked to anti-RFP) (Prior *et al*. 2003b; Zhou *et al*. 2012, 2014).

  (A) Open an EM image in ImageJ, assign the *x* and *y* coordinates of each small and large gold particle within a selected 1 μm^2^ area, and save them in separate files (Fig. 2B).
  (B) How each big gold particle (6 nm) co-clusters with all small particles (2 nm) and how each small gold co-localizes with all big gold particles are analyzed using a variation of the Ripley’s *K*-function, bivariate *K*-functions (Eqs. 3–6):
    3 $$K_{\text{biv}} \left( r \right)\, = \,\left( {n_{\text{b}} \, + \,n_{\text{s}} } \right)^{ - 1} \left[ {n_{\text{b}} K_{\text{sb}} \left( r \right)\, + \,n_{\text{s}} K_{\text{bs}} \left( r \right)} \right]$$
    4 $$K_{\text{bs}} \left( r \right) = \frac{A}{{n_{\text{b}} n_{\text{s}} }}\mathop \sum \limits_{i = 1}^{{n_{\text{b}} }} \mathop \sum \limits_{j = 1}^{{n_{\text{s}} }} w_{ij} 1(\Vert x_{i} - x_{j} \Vert \le r)$$
    5 $$K_{\text{sb}} \left( r \right) = \frac{A}{{n_{\text{b}} n_{\text{s}} }}\mathop \sum \limits_{i = 1}^{{n_{\text{s}} }} \mathop \sum \limits_{j = 1}^{{n_{\text{b}} }} w_{ij} 1(\Vert x_{i} - x_{j} \Vert \le r)$$
    6 $$L_{\text{biv}} \left( r \right) - r = \sqrt {\frac{{K_{\text{biv}} \left( r \right)}}{\pi }} - r$$
    where *K*_bs_(*r*) depicts the distribution of the 6-nm (“b” means big) gold particles with respect to each 2-nm (“s” means small) gold particle and reciprocally *K*_sb_(*r*) characterizes the distribution of small gold particles with respect to each big gold particle. *K*_biv_(*r*) estimator combines the both *K*_bs_(*r*) and *K*_sb_(*r*) to generate a single function, where *n*_b_ means number of big (6-nm) gold particles and *n*_s_ means number of small (2-nm) gold particles and other notation is as in Eq. 1 and 2 (Fig. 2C). Monte Carlo simulations then yield a 95% confidence interval, which further standardizes *L*_biv_(*r*) − *r*. We then use a term, LBI, as a defined integral of the standardized *L*_biv_(*r*) − *r* function to summarize statistic of extent of co-localization (Zhou *et al*. 2014):
    7 $$LBI = \mathop \smallint \nolimits_{10}^{110} {\text{Std}} L_{\text{biv}} \left( r \right) - r \cdot {\text{d}}r$$
    For each condition, a minimum of 15–20 PM sheets are imaged and analyzed. Bootstrap tests used in the univariate analysis are also used to evaluate the statistical significance of differences between two bivariate data sets.

## Discussion

The spatial distribution of the membrane proteins and the lipids on/in the PM is critical to their activities and cell signaling/function. Many speculate heterogeneity in the PM, where the proteins and the lipids laterally segregate into specialized domains, which play essential roles in the function of these proteins, *i.e.*, their ability to be recruited to the activation sites and be activated, their ability to recruit downstream effectors, and their ability to establish signaling network with other components in the PM (Hancock 2003, 2006; Nan *et al*. 2013; Simons and Toomre 2000; Zhou and Hancock 2015). The traditional fluorescence microscopy can qualitatively pinpoint the location of excess localization, such as the observation of puncta formation in cells. However, these techniques do not have high enough spatial resolution to yield quantitative measurement of the extent of the lateral segregation because of the optical resolution limit of 200 nm. FRET-based techniques are powerful methodologies in measuring dimerization/oligomerization within a distance less than 10 nm (Meyer *et al*. 2006). The effective distance is limited to 10 nm because the fluorescence energy transfer only occurs when the donors and the acceptors are less than 10 nm apart. Additionally, traditional techniques for measuring membrane recruitment, such as immuno-blotting, cannot distinguish between PM fractions versus endomembrane fractions.

The EM-spatial mapping, on the other hand, is a highly quantitative analysis to mathematically characterize the ability of the membrane proteins or the lipids to laterally distribute on the PM. This technique has a length scale from 5 to 240 nm, providing crucial information on the nano-scale spatial organization of the PM components over a long-distance range. The quantitative parameters, such as clustered fraction and number of proteins per cluster, yield critical information on the extent of oligomerization and the potential role of oligomers in signal transmission. Because our protocol is optimized to attached intact PM sheets, but not other endomembrane compartments, to the EM grids, the EM-spatial analysis effectively quantifies the spatial distribution of the membrane proteins exclusively on the intact PM, without the background from endomembranes. The bivariate co-clustering analysis mathematically examines the ability of the two populations of species to co-localize on the PM, as well as substrate-effector recruitment on the PM. This co-localization methodology characterizes oligomerization on a length scale (8–240 nm) that cannot be obtained via FRET-based imaging methods, as the energy transfer between FRET donor and acceptor is limited to < 10 nm. We have used the bivariate analysis extensively to map the lipid composition of various Ras nanoclusters.

The primary weakness of the EM-spatial mapping methodology is that we need to flatten and fix cells to attach the apical/basolateral PM onto the EM grids for imaging. Although the results obtained via the EM-spatial mapping technique have been validated by traditional methods using intact/live cells, such as FLIM–FRET, FRAP, FCS, and single particle tracking, and molecular simulations from our group and other investigators, the EM technique still does not reflect the most native state of cells. The EM-spatial mapping technique also relies on the use of fluorescently tagged proteins. It is still not clear how tagging a GFP molecule could potentially influence the lateral movement/distribution of a protein. Thus, it is critical to validate that the fluorescent tag does not influence the function of the proteins tested. The reliance of the EM-spatial analysis on the over-expression of GFP-tagged proteins/peptides also presents issues because an increase in the protein level could potentially influence the extent of nanoclustering of the proteins on the PM. Interestingly, early studies have shown convincing evidence that membrane proteins, especially Ras small GTPases, maintain constant clustered fraction and the extent of the nanoclustering over many orders of magnitude of the expression level (Tian *et al*. 2007).

When mapping the lipid distribution in the PM, we mainly use the GFP-tagged lipid-binding domains, such as the well-established C2 and PH domains, to track the location of lipids in the PM. However, the over-expression of the lipid-binding domains could potentially influence cell signaling by competitive binding to the lipids, thus effectively quenching the available concentrations of these lipids. This could especially become problematic when studying the lipid co-localization with the proteins that have the same lipid-binding domains. Thus, when studying the lipid composition of the membrane proteins, proper control experiments should be performed. For instance, activities of the involved signaling cascades should be checked with/without the over-expression of certain lipid-binding domain to ensure that the over-expressed lipid-binding domain does not artificially influence the localization and the activities of the membrane proteins by competing with the proteins for any lipid. The co-localization between the lipids and the membrane protein should also be validated using the fluorescent lipids in a FRET-based technique.

Detailed information of the reagents used is listed in Table 3.

**Table 3:** Reagents utilized in this work

Reagent	Company	Cat#
Pioloform	TED PELLA	19244
Choroform	Electron Microscopy Sciences (EMS)	12550
Poly-l-lysine	Sigma	P8920-100ML
EM copper grids	EMS	G200-CU
Trisodium citrate	Sigma	C3674-100G
Tannic acid	EMS	21710
Potassium carbonate	Sigma	P5833-500G
Potassium acetate	Sigma	P1190-1KG
Glycerol	Sigma	G55-1L
Paraformaldehyde	EMS	1570
Glutaraldehyde	EMS	16220
Fish gelatin	Sigma	G7765-250ML
BSA	Sigma	A6003-100G
Gold chloride	Sigma	254169-5G
